# SARS-CoV-2 raw wastewater surveillance from student residences on an urban university campus

**DOI:** 10.3389/fmicb.2023.1101205

**Published:** 2023-02-09

**Authors:** K. T. Ash, Y. Li, I. Alamilla, D. C. Joyner, D. E. Williams, P. J. McKay, B. M. Green, C. Iler, S. E. DeBlander, C. M. North, F. Kara-Murdoch, C. M. Swift, T. C. Hazen

**Affiliations:** ^1^Biosciences Division, Oak Ridge National Laboratory, Oak Ridge, TN, United States; ^2^Department of Civil and Environmental Sciences, University of Tennessee, Knoxville, TN, United States; ^3^Student Health Center, University of Tennessee, Knoxville, TN, United States; ^4^Center for Environmental Biotechnology, University of Tennessee, Knoxville, TN, United States; ^5^Department of Microbiology, University of Tennessee, Knoxville, TN, United States; ^6^Facilities Services Department, University of Tennessee, Knoxville, TN, United States; ^7^College of Natural Science, Michigan State University, East Lansing, MI, United States; ^8^Battelle Memorial Institute, Columbus, OH, United States; ^9^Department of Earth and Planetary Sciences, University of Tennessee, Knoxville, TN, United States; ^10^Institute for a Secure and Sustainable Environment, University of Tennessee, Knoxville, TN, United States

**Keywords:** SARS-CoV-2, COVID-19, wastewater, RT-qPCR, raw wastewater

## Abstract

The COVID-19 pandemic brought about an urgent need to monitor the community prevalence of infection and detect the presence of SARS-CoV-2. Testing individual people is the most reliable method to measure the spread of the virus in any given community, but it is also the most expensive and time-consuming. Wastewater-based epidemiology (WBE) has been used since the 1960s when scientists implemented monitoring to measure the effectiveness of the Polio vaccine. Since then, WBE has been used to monitor populations for various pathogens, drugs, and pollutants. In August 2020, the University of Tennessee-Knoxville implemented a SARS-CoV-2 surveillance program that began with raw wastewater surveillance of the student residence buildings on campus, the results of which were shared with another lab group on campus that oversaw the pooled saliva testing of students. Sample collection began at 8 am, and the final RT-qPCR results were obtained by midnight. The previous day’s results were presented to the campus administrators and the Student Health Center at 8 am the following morning. The buildings surveyed included all campus dormitories, fraternities, and sororities, 46 buildings in all representing an on-campus community of over 8,000 students. The WBE surveillance relied upon early morning “grab” samples and 24-h composite sampling. Because we only had three Hach AS950 Portable Peristaltic Sampler units, we reserved 24-h composite sampling for the dormitories with the highest population of students. Samples were pasteurized, and heavy sediment was centrifuged and filtered out, followed by a virus concentration step before RNA extraction. Each sample was tested by RT-qPCR for the presence of SARS-CoV-2, using the CDC primers for N Capsid targets N1 and N3. The subsequent pooled saliva tests from sections of each building allowed lower costs and minimized the total number of individual verification tests that needed to be analyzed by the Student Health Center. Our WBE results matched the trend of the on-campus cases reported by the student health center. The highest concentration of genomic copies detected in one sample was 5.06 × 10^7^ copies/L. Raw wastewater-based epidemiology is an efficient, economical, fast, and non-invasive method to monitor a large community for a single pathogen or multiple pathogen targets.

## Introduction

In the fall semester of 2020, the University of Tennessee-Knoxville put into place numerous precautions meant to limit the spread of the SARS-CoV-2 virus among students on campus. The new limitations included a hybrid approach of online and “in-person” classes, reduced capacity in dormitories, contact tracing, isolation of infected individuals, saliva testing, and wastewater-based surveillance for SARS-CoV-2 of the Knoxville campus dormitories and Greek village. Wastewater surveillance for SARS-CoV-2 at a university was first reported when the University of Arizona announced they detected SARS-CoV-2 in a wastewater sample from a campus dormitory in April 2020 (Betancourt et al., 2021). As of 6 December 2022, 283 universities in 70 countries worldwide have been involved with wastewater surveillance since the pandemic’s beginning (Naughton et al., 2021; COVIDPoops19_Dashboard, 2022). Wastewater surveillance, or wastewater-based epidemiology, has been heralded as a testing procedure that can predict where outbreaks will occur. Multiple studies indicate that wastewater surveillance results can predict cases and outbreaks where individual clinical testing has yet to detect them (Betancourt et al., 2021; Bivins and Bibby, 2021; Gibas et al., 2021; Karthikeyan et al., 2021; Scott et al., 2021; Fahrenfeld et al., 2022). Because we know the exact source of the sample, the response can be rapid by directing the public health officials to specific residences of concern based on the wastewater analysis results. Direct-building sampling of raw wastewater has become the most utilized WBE sampling method employed in monitoring programs on university campuses (Harris-Lovett et al., 2021). WBE can predict an onset of disease with a lead time of up to 4 days (Bibby et al., 2021). When individual testing is easily accessible and available, the WBE results would not be as sensitive or predictive as the clinical results. However, when individual testing is limited, the WBE results become a leading indicator of impending cases and outbreaks (Fahrenfeld et al., 2022).

Additionally, wastewater surveillance is a more affordable option in comparison to individual clinical testing. The cost would be a deciding factor for communities and organizations with limited funds available for the surveillance of SARS-CoV-2 or other pathogens by RT-qPCR. One study found that testing students individually, over 4 months, amounted to $338,000. During that same timeframe, the wastewater surveillance cost just $6,042, only 1.7% of the cost of the individual testing (Wright et al., 2022). With cost in mind, researchers could use the WBE results to deploy targeted individual testing when potential problems are identified, thereby using individual clinical testing more efficiently (Wright et al., 2022).

## Materials and methods

**Figure 1:**
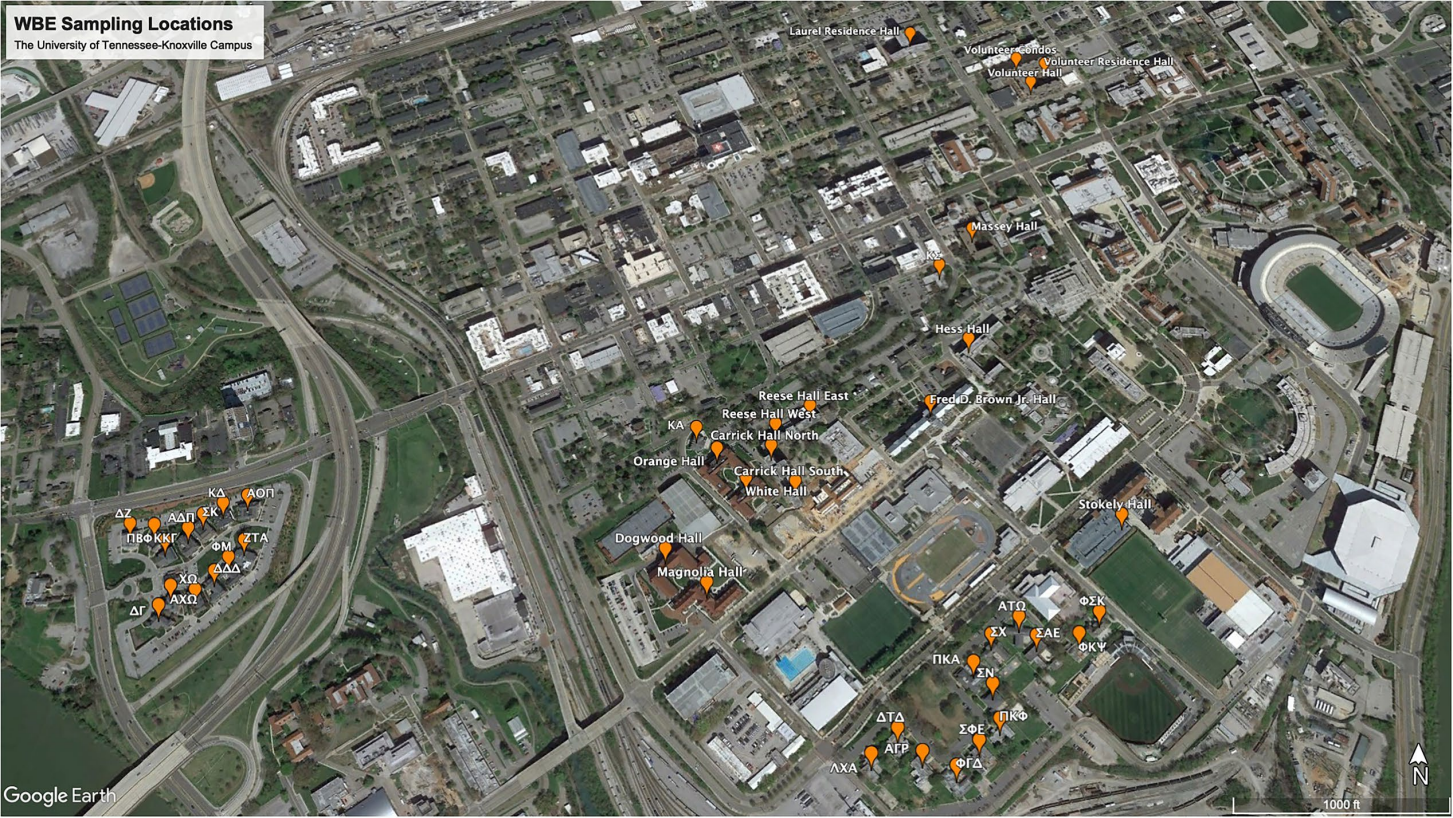
Map of the WBE sampling locations on the University of Tennessee-Knoxville campus.

Wastewater samples were collected from 46 residence halls at the University of Tennessee-Knoxville Campus (Figure 1), commencing in August 2020 and concluding in October 2021. Our sampling routine consisted of testing each of the 46 buildings once every week, with sample collection and analysis divided up between Monday and Thursday. Friday was reserved for cleaning, preparation of reagents, and other miscellaneous needs. Due to the supply chain plastic shortage and logistics of setting up a new lab, our first whole week of sampling began on Monday, 14 September 2020. Samples were collected directly from the building by a sampling valve or the sewer manhole outside the building. Most buildings were sampled *via* a “grab” sample from either a dipping cup from the sewer manhole or a spigot on the wastewater system in the basement of the building. For the buildings with the highest student populations, samples were collected every hour for 24 h, once weekly, with a 24-h Hach AS950 Portable Peristaltic Sampler (Hach, Loveland, CO, United States). To adequately test all the samples collected over 24 h, we would combine the samples into four groups of 6, representing the morning (6—11 am), afternoon (12—5 pm), evening (6—11 pm), and late-night hours (12—5 am). We grouped the samples in this manner because the samples collected during the late-night hours were often less than 50 mL in volume, which is the minimum volume we needed to test the sample.

**Figure 2:**
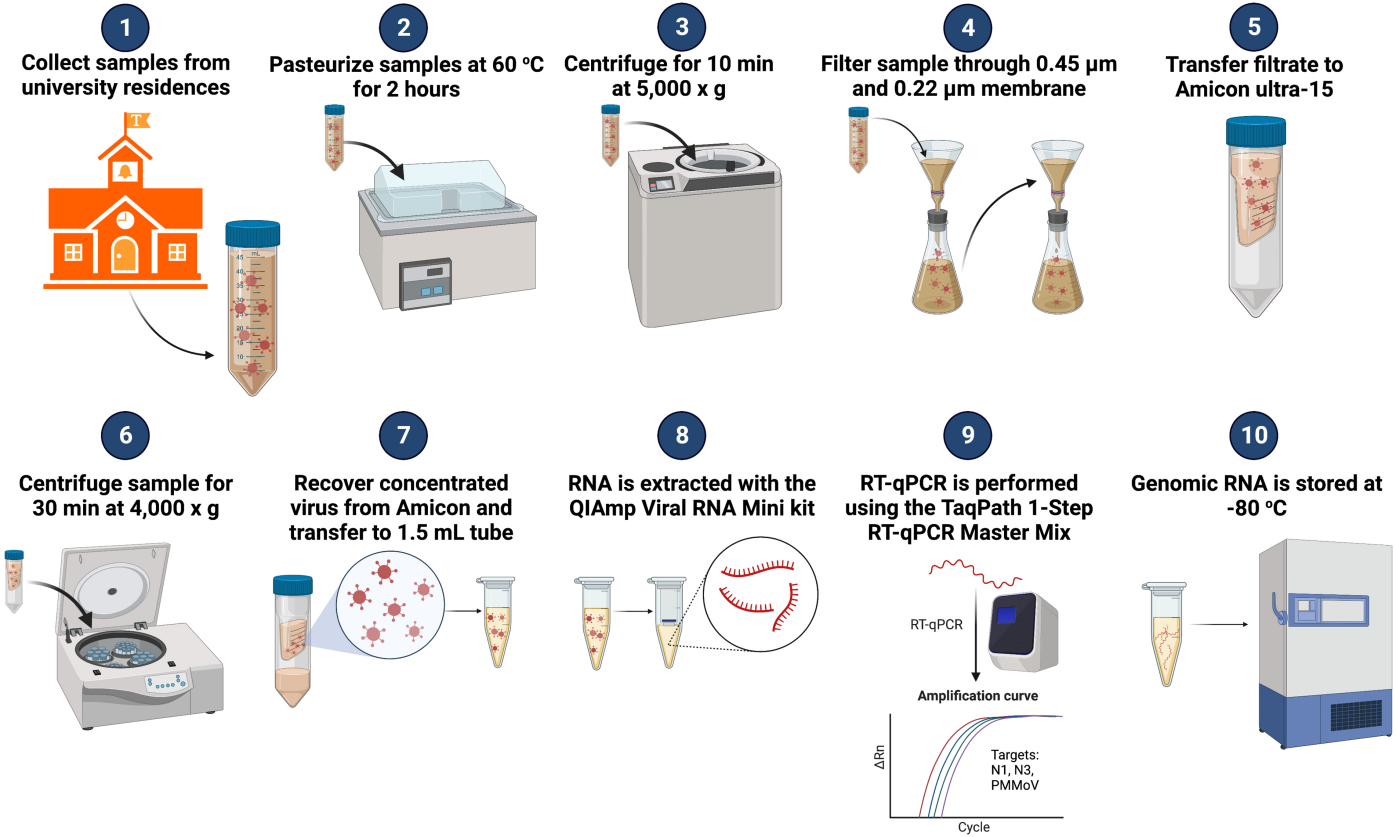
Diagram of WBE workflow for SARS-CoV-2 surveillance on the University of Tennessee-Knoxville campus. Adapted from “Quantifying SARS-CoV-2 Virions in City Wastewater,” by BioRender.com (2022). Available at: https://app.biorender.com/biorender-templates.

After the samples were collected and returned to the lab (Figure 2), the bottles were sprayed with 70% EtOH and allowed to sit for 1 min before being loaded into a water bath where the samples were pasteurized for 2 h at 60°C (Wu et al., 2020). After pasteurization, 50 ml of the sample was transferred to a 50 mL conical centrifuge tube. The 50 mL sample was centrifuged in a fixed-angle rotor for 10 min at 5,000 × *g* to remove heavy sediment from the sample. The supernatant was then filtered through a 10 μm pore diameter Mixed Cellulose Ester (MCE) membrane filter (EMD Millipore, Burlington, MA). The filtrate was collected and filtered through a 0.45 and a 0.22 μm pore diameter (MCE) membrane filter (EMD Millipore, Burlington, MA). The 0.45 and 0.22 μm pore diameter filters were collected and frozen for 16S rRNA analysis. Filtrate (15 ml) was transferred to an Amicon Ultra-15 filtration device (EMD Millipore, Burlington, MA) and centrifuged in a swing-arm rotor for 30 min at 4,000 × *g* to concentrate the viral particles. Approximately, 150—300 μL of the concentrated sample was recovered from the Amicon Ultra-15, and 150 μL was transferred to a 1.5 mL microcentrifuge tube for RNA extraction. The RNA was extracted from the concentrate using the Qiagen QIAamp Viral RNA Mini kit, with carrier RNA, according to the manufacturer’s protocol (Qiagen, Valencia, CA). The extracted RNA was eluted from the QIAamp Mini column in 70 μl buffer AVE. Each sample was tested for the presence of SARS-CoV-2 utilizing Reverse Transcriptase—qPCR (RT-qPCR) with the CDC N1 and N3 primers. These primers are qPCR assays specific for distinct regions of the Nucleocapsid gene of the SARS-CoV-2 genome and provide an estimate of the number of Nucleocapsid gene copies in a test sample (Lu et al., 2020). Initially, the CDC N2 primer was a part of our analysis program, but it consistently underperformed compared to the N1 and N3 primers (i.e., higher Cq value; negative result when sample tested positive with N1 and N3). With consultation from colleagues at the University of Maryland Institute of Genome Sciences, which already had testing programs up and running, we settled on using CDC primer/probe assays N1 and N3 for our WBE sampling analysis. The Pepper Mild Mottle Virus (PMMoV) assay was included in our diagnostic panel to serve as an internal positive control (IPC; Wu et al., 2020). The Pepper Mild Mottle Virus is the most abundant RNA virus in human feces (Zhang et al., 2006). The PMMoV virus is highly stable and has been observed to retain its ability to infect plants after passing through the human digestive tract (Zhang et al., 2006; Rosario et al., 2009; Kitajima et al., 2018). Because the presence of the PMMoV virus in human waste is dietary dependent, the abundance of the virus would be more stable and found in almost all individuals. In contrast, a human pathogen is only found in afflicted individuals at detectable levels (Rosario et al., 2009).

Following the CDC recommendations, the RT-qPCR reaction for assays N1 and N3 (Table 1) were the same regarding reagent concentration and volume. Reactions consisted of 1 μl (10 μM Forward Primer), 1 μl (10 μM Reverse Primer), 0.25 μl (10 μM Probe), 5 μl (TaqPath 1-Step RT-qPCR Master Mix), CG with Rox (Applied Biosystems, Waltham, MA, United States), 7.75 μl (dH_2_O), and 5 μl (RNA Template). The analysis was performed on a QuantStudio 7 Pro (Applied Biosystems, Waltham, MA, United States). The thermocycling conditions for N1 and N3 were the following. Step 1: 2 min @ 25°C, Step 2: 15 min @ 50°C, Step 3: 2 min @ 95°C, Step 4: 3 s @ 95°C, and Step 5: 30 s @ 55°C. Steps 4 and 5 are repeated for 45 cycles (Lu et al., 2020). The RT-qPCR reaction for the PMMoV virus followed previously published conditions (Zhang et al., 2006; Haramoto et al., 2013). Reactions consisted of 1.8 μl (10 μM PMMV-FP1-rev), 1.8 μl (10 μM PMMV-RP1), 0.5 μl (10 μM PMMV-Probe1), 5 μl (TaqPath 1-Step RT-qPCR Master Mix, CG with Rox), 8.9 μl (dH_2_O), and 2 μl (RNA Template). Thermocycling conditions for the PMMoV assay were the following. Step 1: 2 min @ 25°C, Step 2: 15 min @ 50°C, Step 3: 2 min @ 95°C, Step 4: 30 s @ 95°C, and Step 5: 1 min @ 60°C. Steps 4 and 5 are repeated for 40 cycles.

**Table 1:** RT-qPCR primer and probe sequences.

Target	Primer designation	Primer sequence
N1	Forward	5′-GACCCCAAAATCAGCGAAAT-3′
	Reverse	5′-TCTGGTTACTGCCAGTTGAATCTG-3′
	Probe	5′-FAM-ACCCCGCATTACGTTTGGTGGA CC-BHQ1-3′
N3	Forward	5′-GGGAGCCTTGAATACACCAAAA-3′
	Reverse	5′-TGTAGCACGATTGCAGCATTG-3′
	Probe	5′-FAM-AYCACATTGGCACCCGCAATC CTG-BHQ1-3′
PMMoV	PMMV-FP1-rev	5′-GAGTGGTTTGACCTTAACGTTTGA-3′
	PMMV-RP1	5′-TTGTCGGTTGCAATGCAAGT-3′
	PMMV-Probe1	5′-FAM-CCTACCGAAGCAAATG-MGB-NFQ-3′

All primers and probes were ordered from IDT (Coralville, IA; Zhang et al., 2006; Haramoto et al., 2013; Lu et al., 2020).

Genomic RNA isolated from SARS-CoV-2 strain 2019-nCoV/USA-WA1/2020 was obtained from American Type Culture Collection (Cat #: VR-1986D; ATCC, Manassas, VA) and prepared in a serial dilution to be used in the construction of a 6-fold standard curve for assay N1 and N3. A gBlock from IDT (Coralville, IA) was used to create the standard curve for the PMMoV assay. The standard curves for all assays began with a concentration of 10^6^ copies/μL and were serially diluted in a 1/10 ratio to 10^1^ copies/μL. Each concentration was tested, in triplicate, by RT-qPCR. The Limit of Quantification was determined as the concentration at which all three reactions of the same concentration are positive and stay within a Cq value of 0.8 of each other. The standard curve for N1 had a percent efficiency of 94.669% with an *R*^2^ value of 1. The percent efficiency of the N3 assay (~90%) was less than that of the N1 assay; therefore, the copy number values for N1 were the only values reported for the positive samples. Each sample was tested separately for the N1 and N3 assay in triplicate reactions and was only recorded as a positive hit for that assay target if two of the three triplicate reactions were positive. Both N1 and N3 had to be recorded as positive hits for the final result to be recorded as a sample positive for the presence of SARS-CoV-2. The PMMoV assay standard curve had a percent efficiency of 100.109% and was prepared using a gBlock (IDT, Coralville, IA) of the target region. The baseline recovery efficiency for our workflow was determined by using an aliquot of a heat-inactivated SARS-CoV-2 lysate (Cat #: VR-1986HK) obtained from the American Type Culture Collection (ATCC, Manassas, VA). The aliquot was diluted 1/10 and spiked into 50 mL of sterile water. This spike sample was then processed through our entire workflow, and RT-qPCR Cq values were compared to the Cq values of a 1/10 dilution that was only processed through the RNA extraction procedure and RT-qPCR. The percent recovery was determined to be ~0.8% when the sample matrix was sterile water. Betancourt et al. reported the results of matrix spike assays to yield an average recovery of 14 ± 16%, and that the variation in the recovery efficiency is due to the complexity of the environmental matrix (Hellmer et al., 2014; Michael-Kordatou et al., 2020; Betancourt et al., 2021). Similar to Betancourt et al., our results were not normalized to the recovery efficiency of the testing method. In future studies, we recommend injecting a positive control sample that serves as a tool to measure the recovery efficiency of every sample collected.

## Results

In the project’s initial phase, the Facilities Department supplied us with a list of buildings on campus where students resided (Figure 1). This list included all sororities, fraternities, and dormitories. The sororities and fraternities were accessed by a sewer manhole, allowing for collection from the waste stream directly associated with the building. The dormitories were sampled through a direct sample valve located in the basement of the buildings. Dormitory 43 was the exception for the dormitories because it was always sampled *via* the sewer manhole. The PMMoV assay was applied to 1,243 samples in all, and the average PMMoV genomic copies detected was 1.74 × 10^8^ copies/L. Of the 1,243 samples tested for PMMoV, only six returned as “undetectable.” These six samples were also negative for SARS-CoV-2.

**Figure 3:**
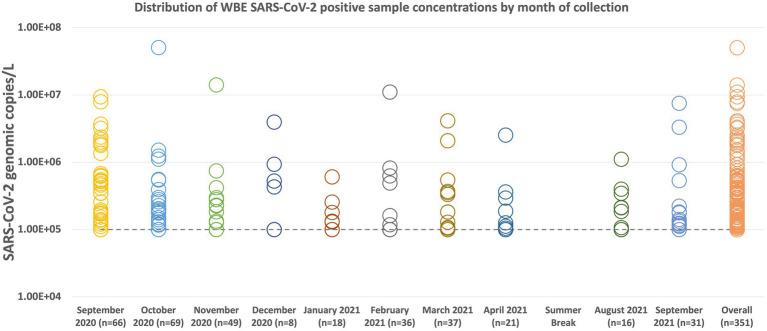
Distribution plot of all SARS-CoV-2 positive sample concentrations by collection month. Samples below our limit of quantification of 10 copies/μL, but were determined to be positive for the presence of SARS-CoV-2, were labeled as 10 copies/μL for presentation purposes. When converted to copies/L, the limit of quantification is 1 × 10^5^ copies/L (represented by the dashed line on the graph). The average concentration for the entire group of positive samples (Overall *n* = 351) is 5.45 × 10^5^ genomic copies. Of the 351 positive samples collected, 231 were at or below our limit of quantification. During the summer break of 2021, samples were only collected from three dormitories, the results of which are not displayed here.

Figure 3 summarizes the WBE sampling results by month of collection. The sample with the highest concentration was collected in October with a concentration of 5.06 × 10^7^ genomic copies/L. The average copies/L of all the samples is 5.45 × 10^5^ genomic copies/L. December 2020 had the lowest amount of positive samples (*n* = 8) collected due to the end of the semester/holiday break. October 2020 was the month with the highest number of samples collected and the number of positive samples detected. For all of the collection months, at least half of the positive samples were at or below the level of quantification. Only September 2020 had a median value (1.06 × 10^5^ copies/L) that was higher than our limit of quantification of 1 × 10^5^ copies/L. October 2020 had the highest average concentration at 9.23 × 10^5^ copies/L.

**Figure 4:**
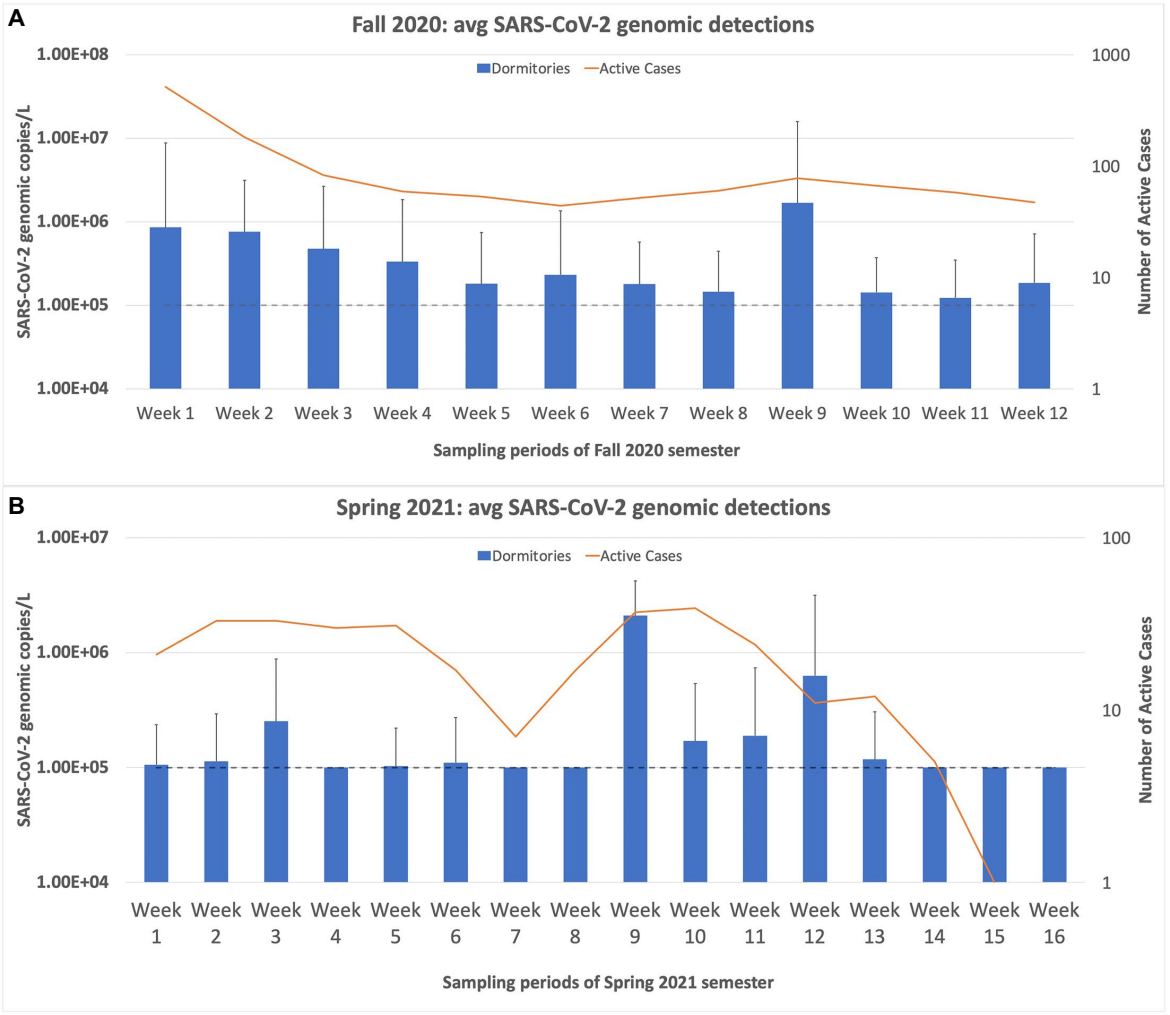
Weekly sampling results for the university dormitories plotted against the active cases in the dormitories, as reported by the student health center on campus. **(A)** displays the sampling results for the Fall 2020 semester, and **(B)** displays the results for the Spring semester of 2021. The dashed line in both charts represents the limit of quantification for the N1 RT-qPCR assay. Any positive samples deemed “below quantifiable levels” are listed at 10 copies/μL. The error bars represent the maximum range of the positive sample concentrations above the average. Spearman’s rank correlation was computed to determine the relationship between the average SARS-CoV-2 copies/L detected in the wastewater samples of the dormitories and the active cases reported by the Student Health Center. There was a moderately positive correlation between the two variables, *r*(25) = [0.653], *p* = [0.0002].

The first week of sampling produced our highest percentage of positive samples (69%; 25 of 36 buildings) in all the weeks of sampling. The following week, our percentage of positive samples dropped to 49%, followed by a steady decline in the number of positives detected. Figure 4 demonstrates that our wastewater sampling results for the dormitories followed a similar trend to that of the active cases on campus, even displaying the drop in cases in May 2021. A Spearman’s rank correlation determined that a moderately positive relationship, *r*(25) = [0.653], *p* = [0.0002], existed between the average SARS-CoV-2 copies/L detected in the wastewater samples of the dormitories and the number of active cases on campus as reported by the Student Health Center (Encyclofstatistics, 2008).

**Figure 5:**
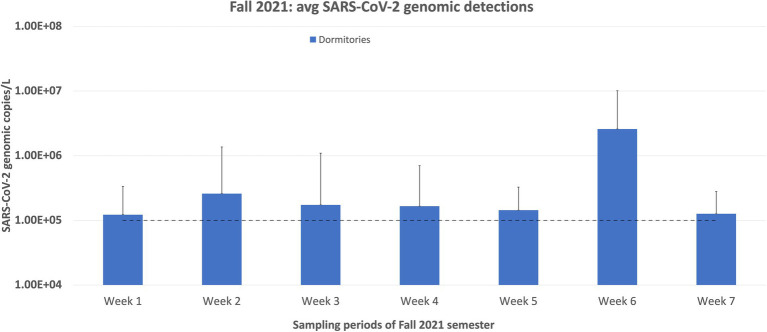
Weekly sampling results for the Fall semester of 2021. This sampling period was deemed necessary due to the rise of the Delta variant, which caused an increase in positive cases following the summer of 2021. During this time, only the dormitories were sampled and not the Greek village because all incoming first-year students were required to live in the dormitories, and most students in the Greek village should have been vaccinated by the Fall of 2021. Therefore, we estimated that the sampling results for the Greek village would be low to non-detectable. Also, students were not required to submit saliva samples for testing during this semester, which is why the active cases are not plotted here. The dashed line represents the limit of quantification for the N1 RT-qPCR assay. Any positive samples deemed “below quantifiable levels” are listed at 10 copies/μL. The error bars represent the maximum range of the positive sample concentrations above the average.

During the fall of 2020 semester, the average copy number of the dormitories consistently stayed above 10^5^ genomic copies/L, for the entire semester. The spring of 2021 semester results showed that in five out of 16 weeks of sampling, the average copy number fell below 10^5^ genomic copies/L. This trend of decreasing average copy numbers in the wastewater led the university administrators to inform us that wastewater monitoring would no longer be necessary. The last sampling day was 2 June 2021. In August 2021, the Student Health Center reported an increasing trend of students who tested positive for SARS-CoV-2, partly due to the rise of the Delta variant. Therefore, university administrators requested that we continue the wastewater surveillance project for the Fall 2021 semester. The sampling resumed on 17 August 2021, and continued until 11 October 2021. Over this ~2-month sampling period, we saw a sharp increase in the average copy number and percentage of positive samples compared to the final results of the Spring 2021 semester. However, this trend quickly declined over the month of September. In the last week of sampling, only two samples were positive, and only one was above the quantification level (Figure 5).

**Figure 6:**
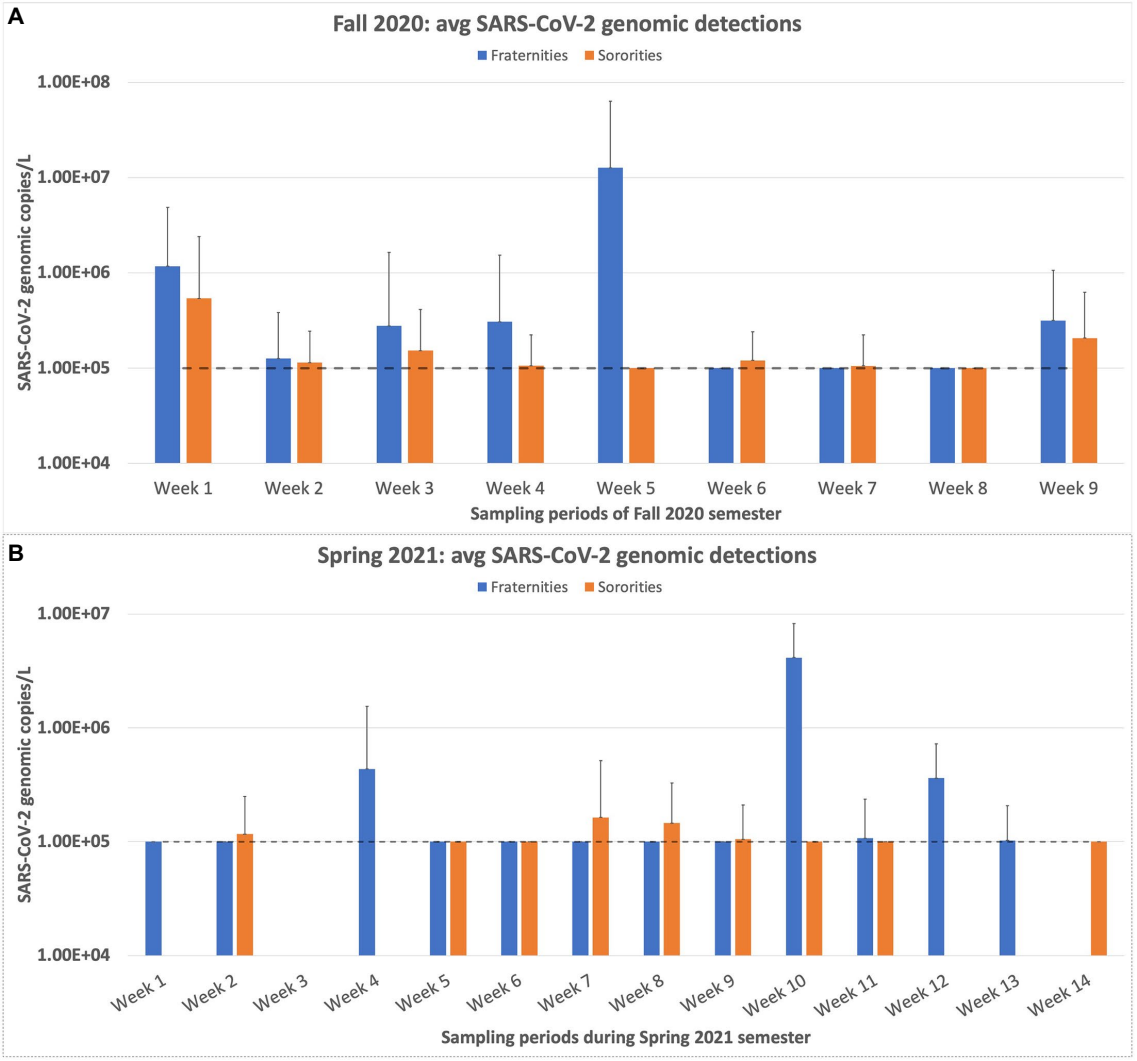
Weekly Sampling results for the residences of the Greek village on campus. **(A)** represents the weekly sampling results of the Fall 2020 semester. **(B)** displays the weekly sampling results for the Spring 2021 semester. The dashed line in both charts represents the limit of quantification for the N1 RT-qPCR assay. Any positive samples deemed “below quantifiable levels” are listed at 10 copies/μl. The error bars represent the maximum range of the positive sample concentrations above the average.

When we examine the results of the buildings individually, a wide variation is observed regarding the number of positive samples detected for each building. Ten buildings accounted for 55% of all the positive samples collected. The total number of samples collected for this group of 10 buildings was only 383, compared to the 994 samples collected from the other 36 buildings during the entire sampling period. Dormitory 38 produced the most positive detections at 29 positive detections out of 42 samples collected, a positive sample rate of 69%. Three buildings, which reside in the Greek village, produced zero positive detections. These buildings were all sampled, at a minimum, for 17 weeks and housed 24 students on average. When sampling first began, the results for the Greek village appeared to be comparable between the fraternities and sororities, though this observation was not statistically significant. However, as the semester progressed, the sampling results became highly variable (Figure 6). Our highest sample came from a fraternity during the second week of October with a copy number of 5.06 × 10^7^ copies/L. The following week, the average copy number for the fraternities fell below detectable levels. Overall, the average genomic copies detected in the dormitories stayed higher and more consistent in comparison to the results of the Greek village samples. The Greek village residences contained significantly fewer students than the dormitories. The average student population of the dormitories was 404 students, with the highest dormitory population at 668 students and the lowest at 229 students. This is in contrast to the student population of the Greek village, which has an average residence of 30 students, with building 22 having the highest population of 50 students.

## Discussion

An anomaly in our results is Dormitory 33. This dormitory was reported to house over 500 students when classes began in August 2020. However, during our entire sampling period, we only detected one positive sample from this dorm. This was quite unusual since, for every other dorm that housed at least 300 students, we collected an average of 17 positive samples. The results from dormitory 33 are more in-line with the results of the dormitories that housed between 200 and 300 students, which had, on average, six positive samples. In our results, dormitory 33 is among the seven dorms with the lowest detections; the other six buildings are found in the Greek village and housed significantly fewer students. The question then becomes why dormitory 33 produced so few positive results.

Dormitory 33 was sampled from a direct valve in the basement of the building. The average level of PMMoV detected in the samples was on par with the other buildings we tested, ranging from 10^5^ to 10^8^ genomic copies/L. The average PMMoV concentrations for all buildings we tested ranged from 10^6^ to 10^9^. Our range of PMMoV concentrations is slightly below the range; a previous study had detected for PMMoV concentrations in raw wastewater (10^7^–10^10^; Kitajima et al., 2018). The PMMoV levels tell us that the samples collected from Dormitory 33 are not being diluted or degraded any more than samples from the other buildings on campus. However, the fact that our average PMMoV range is lower than the range observed by Kitajima et al., indicates that the workflow we employed to process the samples may be hindering our results due to low efficiency. In hindsight, preliminary tests of recovery methods and internal positive controls are essential to find the most efficient method for WBE sampling. Under the emergency circumstances under which this project was started, these aspects were not considered.

Individual saliva results, collected by a separate laboratory on campus, for dormitory 33 indicated that there were students who were positive for SARS-CoV-2 living in dorm 33 and the number of positive saliva samples was on par with the other dormitories of similar size (Data unpublished). More research is required to assess the reasons for the low detection level in wastewater. In hindsight, the use of the 24-h sampling apparatus for this building potentially would have given us the positive samples we could not detect with the grab samples. This idea is supported by a recent study that tested a residence hall on the University of Windsor (Ontario, Canada) campus by grab samples for 7 weeks and had zero detections. However, when the researchers switched to a modified Moore swab approach, every sample they collected from the passive sampling method was positive for the presence of SARS-CoV-2 (Corchis-Scott et al., 2021).

Wastewater surveillance is an efficient, non-invasive method to monitor a community for various drugs, chemicals, and pathogens (Xagoraraki and O’brien, 2020). For example, wastewater surveillance is currently being used to monitor the spread of the emerging pathogens Monkey Pox and the Polio virus (COVIDPoops19_Dashboard, 2022; Editorial, 2022; Wolfe et al., 2022). When WBE is applied to monitor the prevalence of a pathogen in wastewater, it becomes a very effective public health tool. Part of what makes it so valuable is that a single sample can give an estimate of the health of a large community. When you consider that WBE can efficiently direct individual clinical testing and is a fraction of the cost, WBE becomes a method best used in conjunction with individual testing to monitor a given population during an outbreak or pandemic.

Preparations for this project began in June 2020 under a heightened sense of urgency due to the ongoing pandemic. Preparations included hiring the lead postdoc, purchasing necessary equipment, and setting up the diagnostic laboratory. The first preliminary samples were collected in late August 2020, and the first official week of sampling began at the beginning of September 2020. Unfortunately, the urgent need to establish the surveillance program limited our ability to optimize the sampling and analysis methods. In future endeavors of campus wastewater sampling, we recommend using an internal positive control that can be added to each sample to determine the percent recovery. Also, wastewater flow rates for all campus buildings would be beneficial information for researchers to determine the best time for each building to be sampled.

## Data availability statement

The raw data supporting the conclusions of this article will be made available by the authors, without undue reservation.

## Author contributions

KA and TH: writing. IA, DW, PM, and CI: collection. KA, YL, DJ, IA, DW, PM, BG, and SD: lab analysis. FK-M and CS: consulting and collaboration. TH and DJ: project management. CN, TH, KA, and YL: data analysis. All authors contributed to the article and approved the submitted version.

## Funding

Funding for this project was provided by the University of Tennessee Office of Research and Engagement.

## Conflict of interest

FK-M was employed by Battelle Memorial Institute.

The remaining authors declare that the research was conducted without any commercial or financial relationships that could be construed as a potential conflict of interest.

## Publisher’s note

All claims expressed in this article are solely those of the authors and do not necessarily represent those of their affiliated organizations, or those of the publisher, the editors and the reviewers. Any product that may be evaluated in this article, or claim that may be made by its manufacturer, is not guaranteed or endorsed by the publisher.
